# Non-iterative model-based inversion for low channel-count optical ultrasound imaginga)

**DOI:** 10.1121/10.0034450

**Published:** 2024-11-21

**Authors:** Fraser T. Watt, Andreas Hauptmann, Eleanor C. Mackle, Edward Z. Zhang, Paul C. Beard, Erwin J. Alles

**Affiliations:** 1 Department of Medical Physics & Biomedical Engineering, University College London, London, WC1E 6BT, United Kingdom; 2 Wellcome/EPSRC Centre for Interventional and Surgical Sciences, University College London, London, W1W 7TS, United Kingdom; 3 Research Unit of Mathematical Sciences, University of Oulu, Oulu, Finland; 4 Department of Computer Science, University College London, London, United Kingdom

## Abstract

Ultrasound image reconstruction is typically performed using the computationally efficient delay-and-sum algorithm. However, this algorithm is suboptimal for systems of low channel counts, where it causes significant image artefacts. These artefacts can be suppressed through model-based inversion approaches; however, their computational costs typically prohibit real-time implementations. In this work, the emerging optical ultrasound (OpUS) modality is considered, where ultrasound waves are both generated and detected using light. With this modality, imaging probes comprise very low channel counts, resulting in significant image artefacts that limit the imaging dynamic range. However, this low channel counts offer an opportunity for non-iterative (“direct”) model-based inversion (DMI) on modest computational resources available in a typical workstation. When applied to both synthetic and experimental OpUS data, the presented DMI method achieved substantial reduction in image artefacts and noise, improved recovery of image amplitudes, and–after one-off pre-computation of the system matrices–significantly reduced reconstruction time, even in imaging scenarios exhibiting mild spatial inhomogeneity. Whilst here applied to an OpUS imaging system, DMI can be applied to other low channel-count imaging systems, and is therefore expected to achieve better image quality, reduce system complexity, or both, in a wide range of settings.

## I. INTRODUCTION

Ultrasound imaging is a safe, cost-effective, and versatile imaging modality that exhibits excellent soft-tissue contrast and offers good spatial and temporal resolution.^1^ Imaging probes typically contain hundreds of transducer elements, each of which can both transmit and receive ultrasound waves.^1^ The resulting backscatter (“pulse-echo”) radio frequency (RF) data are conventionally reconstructed into images using a “delay-and-sum” (D&S) algorithm, which measures the coherence between pulse-echo signals as detected by multiple transmit–receive detector pairs (“channels”).^1^ The D&S algorithm is highly popular due to its simplicity and numerical efficiency; enabling real-time, video-rate reconstruction even for imaging systems comprising high numbers of elements.^2^

However, the D&S algorithm assumes that patterns of coherence in the data (“actual” signal) constructively add upon reconstruction, and that other components (e.g., noise, clutter) are zero-mean and are hence reconstructed to near-zero values. Whilst this is accurate for systems comprising high numbers of channels, this assumption breaks down in low channel-count scenarios, such as sparse arrays (comprising very few transducer elements), plane wave or photoacoustic imaging [where only a few (or even single) emissions are employed and hence, only limited transmit beamforming is possible^3^] or systems using a single detector (where receive beamforming is not possible).^4^ Consequently, such imaging scenarios tend to suffer from strong image artefacts due to side and grating lobes, measurement noise, inhomogeneous transducer directivity, and geometrical attenuation–resulting in image distortion, clutter, limited dynamic range, and inaccurate image amplitudes, since the D&S algorithm does not consider the physics and signal interactions underlying these artefacts.

Alternative reconstruction algorithms have been devised that are more robust to some or all these sources of artefacts. However, these algorithms either are non-linear [e.g., short-lag spatial coherence (SLSC),^5^ delay-multiply-and-sum (DM&S)^6^] and hence modify the appearance of the actual signal in the image as well as the artefacts, are computationally highly demanding (e.g., minimum-variance beamforming^7^) or require substantial amounts of curated ground-truth training data that can be non-trivial to obtain (e.g., deep learning methods, such as convolutional neural networks applied to either the RF data directly^8^ or as a post-processing step^9^). Whilst such algorithms offer varying levels of image artefact reductions, they still do not consider the physics and signal interactions generating the image artefacts–and hence, cannot address all of the image artefact sources.

In contrast, a branch of image formation techniques exists that can, in principle, capture and correct for most of the artefact-generating principles listed above^10^ (as well as material inhomogeneity and multiple scattering,^11,12^ which is outside the scope of this paper). Rather than directly combining the RF data to form an image, such “model-based inversion” (MI) techniques (or “full waveform inversion” in seismics) instead first numerically predict the RF data generated and detected by a system, using accurate physical numerical models that can incorporate any or all of the artefact-generating mechanisms. Next, these predictions are numerically inverted to match the experimentally observed RF data to the optimal set of physical parameters. These parameters can be converted into an ultrasound image exhibiting substantially reduced artefacts–especially when suitable regularisation or stabilisation is applied.^13^ Such MI approaches have been shown to readily overcome artefacts due to geometrical attenuation, sidelobes, measurement noise artefacts, and transducer element directivity inhomogeneity; however, as grating lobes arising from spatial or temporal undersampling result in ambiguity and distortion of both the modelled and detected RF data, associated artefacts are less effectively suppressed.

Whilst MI methods can be highly effective in reducing image artefacts, or even providing quantitative data, the system matrices involved quickly become prohibitively large to allow for explicit matrix formulation and direct inversion,^14^ especially for densely populated three-dimensional (3D) imaging arrays. Instead, iterative approaches are typically applied that do not require explicit inversion of the system matrix but instead use operators describing the system. Whilst this approach is very successful in a number of applications,^10,15–18^ the required number of iterations precludes real-time implementation in all but the simplest of cases.

Recently, optical ultrasound (OpUS) imaging has emerged as a viable alternative to electronic transducer technology. With OpUS, ultrasound waves are both generated and detected using light; the photoacoustic effect is employed to convert excitation light into broadband ultrasound waves,^19^ and optically resonant ultrasound detectors^20–22^ allow for highly sensitive ultrasound detection using miniature sensors with lateral dimensions down to tens of microns. A number of OpUS imaging paradigms have previously been described, ranging from systems comprising a single OpUS element that is mechanically translated across an aperture,^21,23,24^ to benchtop,^25,26^ freehand,^27,28^ or even non-contact systems^29,30^ capable of real-time, video-rate imaging. However, due to the experimental complexity and cost of OpUS detection systems, each of these paradigms employ just a single OpUS detector, resulting in low RF channel counts ( ≤ca. 200) equal to the number of OpUS sources. Consequently, OpUS imaging systems to date suffer from substantial image artefacts limiting their dynamic range and clinical relevance.

However, the small channel count of OpUS imaging systems actually presents an opportunity to perform non-iterative (“direct”) MI instead, at video-rate and in real-time, implemented on modest hardware. Whilst both iterative and direct model-based inversion (DMI) are well-established in the literature, DMI is ideally suited to OpUS imaging and has not been previously applied to this modality. Note, however, that the concepts can be extended to any scenario exhibiting low RF data channel count. The remainder of this manuscript presents the theory and implementation details of DMI applied to a freehand OpUS imaging system, followed by a number of synthetic and experimental imaging scenarios to characterise the performance of the method.

## II. METHODS

### A. Problem formulation

MI applied to ultrasound imaging aims to solve for the spatiotemporally varying contrast (“reflectivity”) $R(\vec{r},t)$ such that it generates the closest possible match to the measured RF data (the “B-scan”) $B(\vec{r},t)$ when fed to an appropriate forward model. This forward model is based on a mathematical model of the underlying physical phenomena, which predicts the ultrasound field generated by one or more ultrasound sources and detected by a single or more detectors. The predicted B-scan is generated by

$$B(\vec{r},t)=P\left\{R(\vec{r},t)\right\},$$ (1)

where operator P{⋯} applies the forward model to $R(\vec{r},t)$. For linear operators, the forward problem can be written as the matrix-vector multiplication

$$\vec{B}=P\vec{R},$$ (2)

where P is the two-dimensional “system matrix” and $\vec{B}$ and $\vec{R}$ are reshaped into one-dimensional vectors of the B-scan and contrast function, respectively. The system matrix is of dimensions $\left[N_{\text{chan}}\cdot N_{t}\times N_{\text{img}}\right]$, which for large numbers of image pixels $N_{\text{img}}$ and numbers of channels $N_{\text{chan}}$ rapidly becomes impractical to compute and store directly.

For low channel count, however, the memory requirements are modest, meaning that the reflectivity function $\vec{R}$ can be obtained by first explicitly computing the inverse of the system matrix, and subsequently computing

$$\vec{R}\approx {\tilde{P}}^{-1}\vec{B}.$$ (3)

As the system matrix P is highly sparse and rarely square, its inverse either does not exist or cannot be computed exactly due to numerical instability, and instead the least squares solution of Eq. (3) is computed. Regularisation can be applied during inversion to improve on the numerical stability of this inverse matrix. As the matrix-vector multiplication of Eq. (3) can be very efficiently distributed across parallel computation hardware, such as graphical processing units (GPUs), real-time, video-rate applications are readily achieved for imaging scenarios comprising low channel count.

### B. Forward model

The forward operator P can, in principle, be formulated to include any physical phenomenon of note, including multiple scattering, material inhomogeneities, non-linear propagation, and the physical transduction and digitization mechanisms–resulting in a forward model that can predict the detected B-scan in absolute values. However, such highly complex forward operators are non-linear and therefore cannot be implemented by means of a matrix-vector multiplication, such as in Eq. (2), and hence, can only be inverted via iterative means. In addition, the application of forward operators tends to increase in numerical complexity with increasing realism.

In this work, a number of simplifying assumptions will be made to keep the computational load tractable. First, low pressure levels are assumed, and hence, the wave propagation can be assumed linear. Second, the background material is assumed to be homogeneous. Third, the acoustic contrast is assumed to be weak, meaning that multiple scattering can be ignored and the Born approximation is accurate. Fourth, absolute contrast values are not relevant for imaging purposes, and hence, the transduction and digitization processes are not modelled. Finally, the contrast function *R* is assumed to be temporally invariant, i.e., $R(\vec{r},t)\equiv R(\vec{r})$. These assumptions enable modelling of the detected ultrasound field using closed-form, analytical impulse responses, which are computationally efficient and highly accurate. In this work, the FOCUS MATLAB toolbox (MATLAB 2024a, The MathWorks, Natick, MA)^31^ is used in favour of other simulators for its high numerical efficiency and lack of mathematical approximations, resulting in accurate results even in the transducer near-field.^32^

**FIG. 1.**
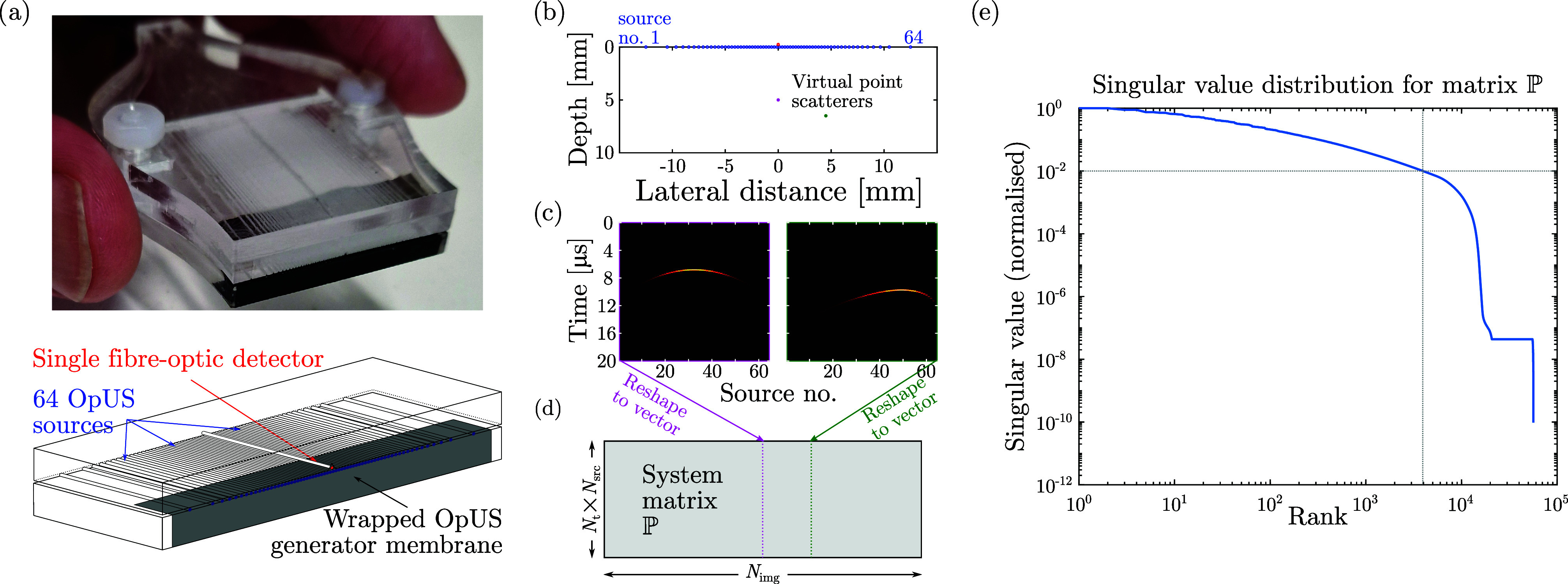
(Color online) **(**a) Photograph (top) and schematic (bottom) of the freehand optical ultrasound (OpUS) imaging probe used and modelled in this paper. The probe comprises 64 irregularly spaced fibre-optic ultrasound sources and a single fibre-optic detector centered within the imaging aperture. (b)–(d) The system matrix P is computed from synthetic data, where a single virtual point scatterer is consecutively placed in all $N_{\text{img}}$ image pixel locations (b), and the resulting pulse-echo A-scan (c) of dimensions $\left[N_{t}\times N_{\text{src}}\right]$) is computed, reshaped, and stored in the corresponding column of the system matrix (d). (e) The normalised singular values obtained after singular value decomposition of system matrix P exhibit a rapid decrease in magnitude with increasing rank, which motivates the application of low-rank decomposition methods. Dashed lines indicate the rank threshold corresponding to a minimum normalised singular value of 1%.

The geometry considered here is that of a freehand OpUS imaging setup^27^ comprising 64 fibre-optic ultrasound sources distributed across a linear aperture and a single, centrally placed fibre-optic ultrasound receiver [Fig. 1(a)]. The sources were modelled as circular piston transducers with diameters matching the core of the optical fibre (200 *μ*m), and subjected to a δ(t) spike distribution as the piston surface velocity source signature. The broadband and omni-directional fibre-optic ultrasound receiver was modelled as a point detector with a flat frequency response using the free-space Green's function [as stated by Cobbold^1^ in Eqs. (2.11) and (2.12), pp. 100]. The system matrix P was then obtained by, for each image pixel, computing the B-scan corresponding to a single mathematical point reflector placed in that pixel location [Figs. 1(b)–1(d)]. Mathematically, this was achieved by convolution of the 64 spatiotemporal impulse responses (modelling the forward propagation from each of the sources to the pixel) with the Green's function (modelling the back-scattering from the pixel to the detector). For computational efficiency, the convolutions and other operations were performed in the frequency domain. For a single scatterer located in image pixel *j*, the pulse-echo time trace corresponding to source element *k* of B-scan B(k,t) was hence computed as

$$B(k,t)=F^{-1}\left\{\hat{H}({\vec{r}}_{\text{img},j}-{\vec{r}}_{\text{src},k},\omega )\cdot \frac{e^{-i\omega \left|{\vec{r}}_{\text{img},j}-{\vec{r}}_{\text{rec}}\right|/c}}{4\pi \left|{\vec{r}}_{\text{img},j}-{\vec{r}}_{\text{rec}}\right|}\cdot i\omega \hat{v}(k,\omega )\right\},$$ (4)

where the coordinates of image pixel *j*, source element *k*, and the single receiver are denoted ${\vec{r}}_{\text{img},j}$, ${\vec{r}}_{\text{src},k}$, and ${\vec{r}}_{\text{rec}}$, respectively. The homogeneous speed of sound is denoted by *c*, and operators $\hat{\cdots }$ and $F^{-1}$ indicate spectral notation and the inverse Fourier transform, respectively. The impulse response for a circular piston transducer $\hat{H}$ was computed using the FOCUS toolbox, the backscattering from the image pixel to the detector was computed using the analytical Green's function, and in this study, the piston surface velocity $\hat{v}=1$ was modelled as a δ(t) spike. See Alles *et al.*^33^ for full implementation details.

### C. Matrix inversion method

The system matrix can be inverted in a variety of ways. However, the singular value decomposition (SVD)^34^ is of particular interest here, as it has a number of interesting properties. The SVD of the system matrix is given by

$$P\approx USV^{T},$$ (5)

where matrices U and V are unitary matrices, S is a diagonal matrix comprising the singular values of P, and operator ^T^ denotes the transpose. Equation (5) is approximate for non-square matrices, as the SVD decomposition was truncated to obtain a square matrix S (equivalent to using the ‘econ’ flag in MATLAB).

Once the SVD is computed, a regularised pseudo-inverse (^†^) of the system matrix is obtained from

$$P^{†}=VS^{†}U^{T},$$ (6)

where diverse regularisation strategies can be applied when computing $S^{†}$ to avoid over-fitting of noise. As the singular values of P rapidly decrease with increasing rank [Fig. 1(e)], the computation of $P^{†}$ can be accelerated without a significant decrease in accuracy^34^ by instead computing a low-rank approximation. This is achieved by truncating U,S, and V to retain only those singular values above a given threshold value. If, in addition, the regularised pseudo-inverse $P^{†}$ is not explicitly computed but instead Eq. (3) is rewritten into the two-stage matrix-vector multiplication

$$\vec{R}\approx P^{†}\vec{B}=V\left[\left(S^{†}U^{T}\right)\vec{B}\right],$$ (7)

the contrast function $\vec{R}$ can be computed at significantly reduced memory and computational load. Where $P^{†}$ is of dimensions $\left[N_{\text{img}}\times N_{\text{chan}}\cdot N_{t}\right]$, sub-matrices V and $S^{†}U^{T}$ are of substantially reduced dimensions $\left[N_{\text{SVD}}\times N_{\text{chan}}\cdot N_{t}\right]$ and $\left[N_{\text{img}}\times N_{\text{SVD}}\right]$, respectively, as the truncation-constrained number of SVD values is substantially decreased and hence, $N_{\text{SVD}}\ll N_{\text{chan}}\cdot N_{t}$ and $N_{\text{SVD}}\ll N_{\text{img}}$. In this work, an empirically determined singular value threshold of $10^{-4}$ was used to balance accuracy and computational requirements, corresponding to *N*_SVD_ = 13 861.

Whilst a variety of regularisation methods are commonly applied, few methods allow for a direct, closed-form implementation.^35^ Here, only two such strategies are considered: truncated SVD (TSVD) and Tikhonov regularisation. With TSVD regularisation, the regularised pseudo-inverse of S is obtained from

$$S_{ii}^{†}(\alpha )=\{\begin{array}{cc} 1/S_{ii} & \text{if}\;S_{ii}\geq \alpha \cdot \text{max}\;(S),\\ 0 & \text{otherwise}, \end{array}$$ (8)

where 0≤α≤1. Conversely, Tikhonov (or L_2_) regularisation is applied by setting

$$S_{ii}^{†}(\alpha )=\frac{S_{ii}}{S_{ii}^{2}+{\left(\alpha \cdot \text{max}\;S\right)}^{2}}.$$ (9)

The system matrix contained $N_{t}\times N_{\text{src}}$ (rows) by $N_{\text{img}}$ (columns) elements, where $N_{t},N_{\text{src}}$, and $N_{\text{img}}$ are the number of time samples, channels, and image pixels, respectively. In this manuscript, all images measure 10 mm (lateral) by 7 mm (axial) at pixel dimensions of 50 by 25 *μ*m, respectively. B-scans are computed or measured using 1004 time samples at a sampling rate of 62.5 MSa/s. Thus, $N_{t}=1004,N_{\text{src}}=64$, and *N*_img_ = 56 481, resulting in a system matrix requiring ca. 13.5 GB of memory when stored in single precision which, whilst substantial, is readily available in modern computers or even GPUs. Computing the SVD required an additional 37.3 GB of memory due to the additional matrices created. However, once the SVD has been computed, the low-rank approximations to matrices $S^{†}U^{T}$ and V require just 3.1 and 2.7 GB of memory, respectively. The computer used in this work featured an Intel Core i9-13900KS (Intel, Santa Clara, CA) central processing unit (CPU), 192 GB of random access memory (RAM), and a NVIDIA RTX 6000 Ada GPU featuring 48 GB of GDDR6 RAM. On this computer, serial computation of the system matrix and SVD, and GPU-accelerated computation of the matrix $S^{†}U^{T}$, required ca. 49 min, 182 min, and 2.14 s, respectively.

### D. Synthetic data

To avoid the “inverse crime,”^36^ where using the same numerical model for both inversion and generation of test data artificially improves the inversion results, synthetic data were generated using the GPU-accelerated version of the *k*-Wave toolbox.^37^ Whereas the forward model used to generate the system matrix was based on closed-form analytical impulse response functions, *k*-Wave utilises a pseudo-spectral time-stepping approach. To limit computation times and enable use of the GPU-accelerated version of *k*-Wave, synthetic data were generated assuming a two-dimensional (2D) geometry, and acoustic reciprocity was applied to reduce the computational complexity to the propagation of the single wave field generated by a point source to 64 finite-sized receivers.

The inherently 3D forward model used for DMI was modified to emulate the 2D *k*-Wave geometry by using “near-infinitely” extended rectangular sources (200 *μ*m wide, 1 cm tall) instead of circular sources. B-scans for each pixel location were computed from coherent summations of the reflections off a “near-infinitely extending” line of scatterers placed orthogonal to the image plane (1 cm tall, 5 *μ*m scatterer separation). By elevationally extending the sources, receiver and scatterers, the scenario was rendered approximately invariant in the elevational direction and hence, mimicked a 2D setting.

To avoid interference from grid edge effects, the spatial grid (28 mm lateral, 12 mm axial, 10 *μ*m grid spacing) used in the *k*-Wave simulations extended beyond the image dimensions, and a substantially smaller temporal step size compared to that of the forward model (1 ns over a 20 *μ*s duration) was used to ensure numerical stability. The imaging aperture was placed one grid point from the edge to avoid interference with an externally located perfectly matched layer.

The OpUS detector was modelled as point-like, and was hence accurately represented by a point source via acoustic reciprocity. This source was positioned centrally within the aperture and modelled as a particle velocity source excited with a tone burst that was spectrally matched to experimental data (four cycles at 11 MHz). The 64 finite-sized circular OpUS sources were approximated as line receivers with a width of 200 *μ*m, which were implemented through coherent summation of the pressure traces observed in 21 adjacent grid points. One additional simulation was performed to simulate and suppress the cross talk from ultrasound waves propagating from the source directly to the receivers (i.e., without scattering off actual contrast), and its results filed for future use. This direct cross talk can be of significantly higher amplitude than the actual pulse-echo signal and thus reduce signal fidelity. Each *k*-Wave simulation required ca. 530 s and 3.2 GB of GPU memory to complete.

Four different synthetic scenarios are considered in this work. For all phantoms, the background medium was modelled as water (speed of sound c=1500 m/s; density ρ=1000 kg/m^3^). First, a single point scatterer was placed centrally in the image at an axial depth of 5 mm by assigning the corresponding grid point a speed of sound of 1570 m/s. Second, a more quantitative phantom comprising three circular regions (diameter: 2 mm; axial depth: 3.5 mm) of varying echogenicity was implemented by assigning each of the corresponding grid points with values randomly generated from the ranges 1497 ≤ *c* ≤ 1503, 1480 ≤ *c* ≤ 1520, and 1440≤c≤1560 m/s. Third, to study the impact on DMI performance of contrast in just speed of sound or just density, a set of two layered phantoms was considered, where each phantom comprised three layers (thickness: 3 mm) of either increasing speed of sound (top to bottom: *c* = 1450, 1500, and 1540 m/s and constant ρ=1000 kg/m^3^) or increasing density (*ρ =* 967, 1000, and 1027 kg/m^3^ and constant c=1500 m/s). Fourth, two phantoms were implemented with geometries mimicking two frames of the experimental data obtained from a needle insertion into a vessel phantom. The first of these frames corresponded to imaging of the vessel phantom in absence of the needle, where the lumen was assigned a speed of sound of c=1570 m/s, the tissue c=1500 m/s, and the whole volume the background density. The second frame corresponded to imaging the same vessel phantom but in the presence of the needle, and used the same spatial geometry and material properties but included a needle approximated as a material with properties c=2000 m/s and ρ=1500 kg/m^3^.

### E. Experimental data

Experimental data were obtained with the setup described in Alles *et al.*^27^ in two imaging scenarios. First, a single, stationary tungsten wire (diameter: 27 *μ*m) was strung orthogonally to the imaging plane at an axial depth of 5 mm, corresponding to a point scatterer in a homogeneous background. Second, a wall-less tissue-mimicking phantom [10% w/w poly(vinyl) alcohol (PVA) cryogel^38^] shaped after a blood vessel was submerged in water, and a 23 G needle was inserted into and retracted from the vessel lumen under continuous imaging at a frame rate of 11 Hz. This second phantom exhibited dynamic changes, material inhomogeneities, and a hyper-echoic needle generating backscatter signal of sufficient amplitude to yield a non-linear response of the OpUS detector–and was chosen to assess the limits of the method.

For both synthetic and experimental data, conventional D&S images were also generated using the GPU-accelerated algorithm presented by Alles and Desjardins.^4^ Whilst more elaborate non-model–based methods (e.g., universal back-projection or time-reversal) exist that could offer similar artefact reduction, such methods are generally not applicable to imaging paradigms utilising just a single detector, like the OpUS modality considered here.

## III. RESULTS

**FIG. 2.**
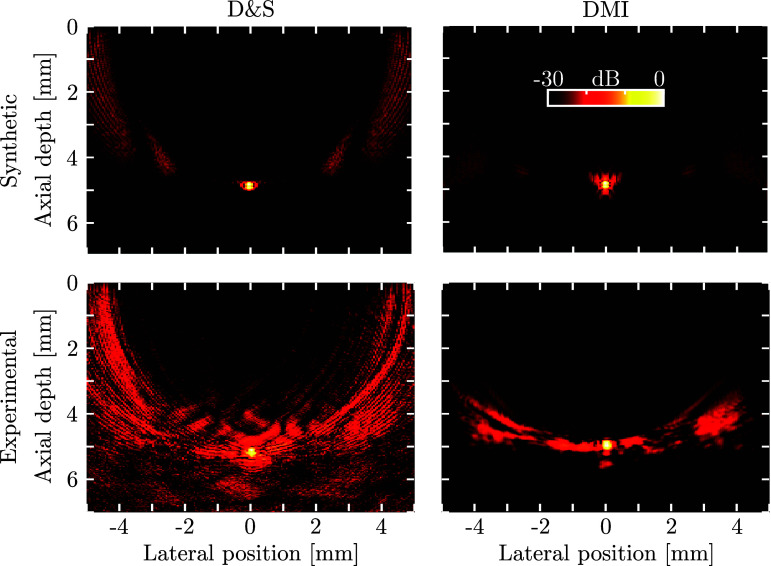
(Color online) D&S (left column) and DMI (right column) for synthetic (top row) and experimental (bottom row) OpUS data acquired or generated for a single point scatterer located at an axial depth of 5 mm. Both DMI images were obtained using Tikhonov regularisation at α=1%. All panels use the same 30 dB dynamic range.

The efficacy of the proposed DMI method in suppressing sidelobes and noise is evident when applied to both synthetic and experimental data for a single point scatterer (Fig. 2). The grating lobes (presented as the “wing-shaped” artefacts located toward the edges of the D&S images) have been substantially reduced by ca. 7 dB in the DMI images, without affecting the axial and lateral resolutions. For the experimental data, an additional decrease in noise level of ca. 15 dB was achieved.

**FIG. 3.**
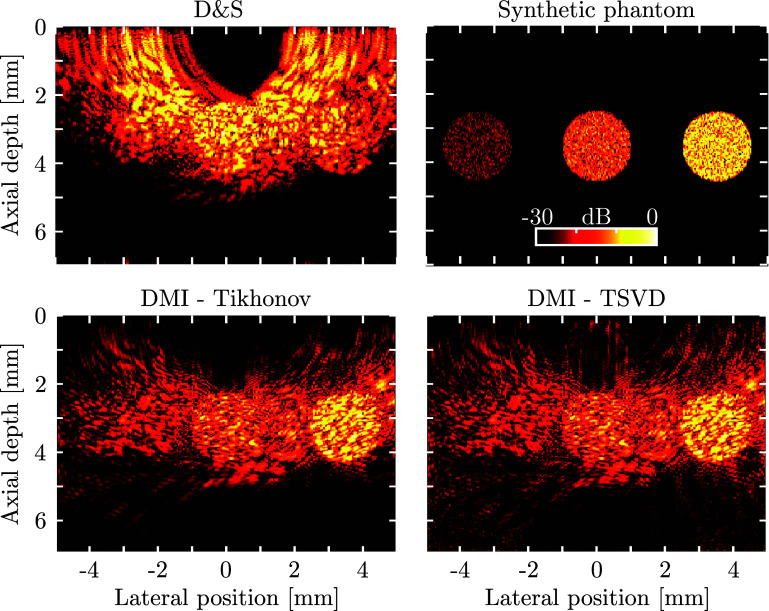
(Color online) Top: D&S (left) image of a numeric phantom (right) comprising three circular inclusions of various echogenicity. Bottom: DMI images for this synthetic phantom using Tikhonov (left) and TSVD (right) regularisation. Both regularisation methods were performed using α=0.1%. All panels use the same 30 dB dynamic range.

Whilst DMI improved on the quality of D&S reconstructed images, the point target considered above effectively constituted a binary scenario, and did not assess the quantitative abilities of the DMI method. Therefore, a second synthetic phantom was considered that comprised several circular regions of varying echogenicity (Fig. 3). For this phantom, the D&S image not only contained significant grating lobe artefacts, but also failed to accurately reproduce the amplitudes of the three regions since the D&S algorithm does not take geometrical attenuation and the source directivity into account. Both DMI images exhibited significantly reduced artefact levels and an increased dynamic range, and in addition, more accurately recovered the amplitudes of the three regions. As previously observed,^33^ especially for larger values of α, Tikhonov regularisation achieved greater suppression of image artefacts than TSVD regularisation, as a greater proportion of singular values contribute to the inverse of the system matrix.

**FIG. 4.**
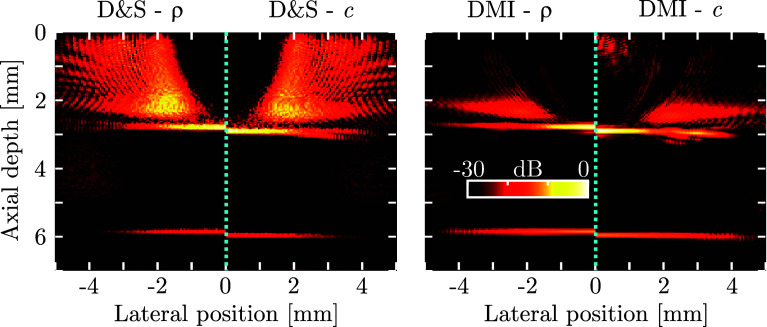
(Color online) Compound D&S (left) and DMI images of a synthetic layered phantom. Each compound image shows the images obtained when either the material density ( ρ, left halves) or the speed of sound (*c*, right halves) varies between the layers. Tikhonov DMI reguarisation was performed using α=1%. All panels use the same 30 dB dynamic range.

The effects of material inhomogeneities are shown in Fig. 4, where compound D&S and DMI images are shown for a simple layered medium. The left half of each image corresponds to a medium where only the density ρ of the medium varies with axial depth but the speed of sound *c* is constant; in the right half of each image, only *c* is depth-dependent whilst keeping ρ constant. As both D&S and DMI algorithms assume a homogeneous speed of sound, spatial distortion of the image is apparent in both cases. However, similar improvements, as shown in Figs. 2 and 3, were observed: the DMI images for both layered phantoms exhibited reduced artefact levels as well as improved recovery of the image quality across the image (most notably for the lower boundary at a depth of ca. 6 mm)—thus confirming the robustness of DMI, even when the requirement of material homogeneity is relaxed.

**FIG. 5.**
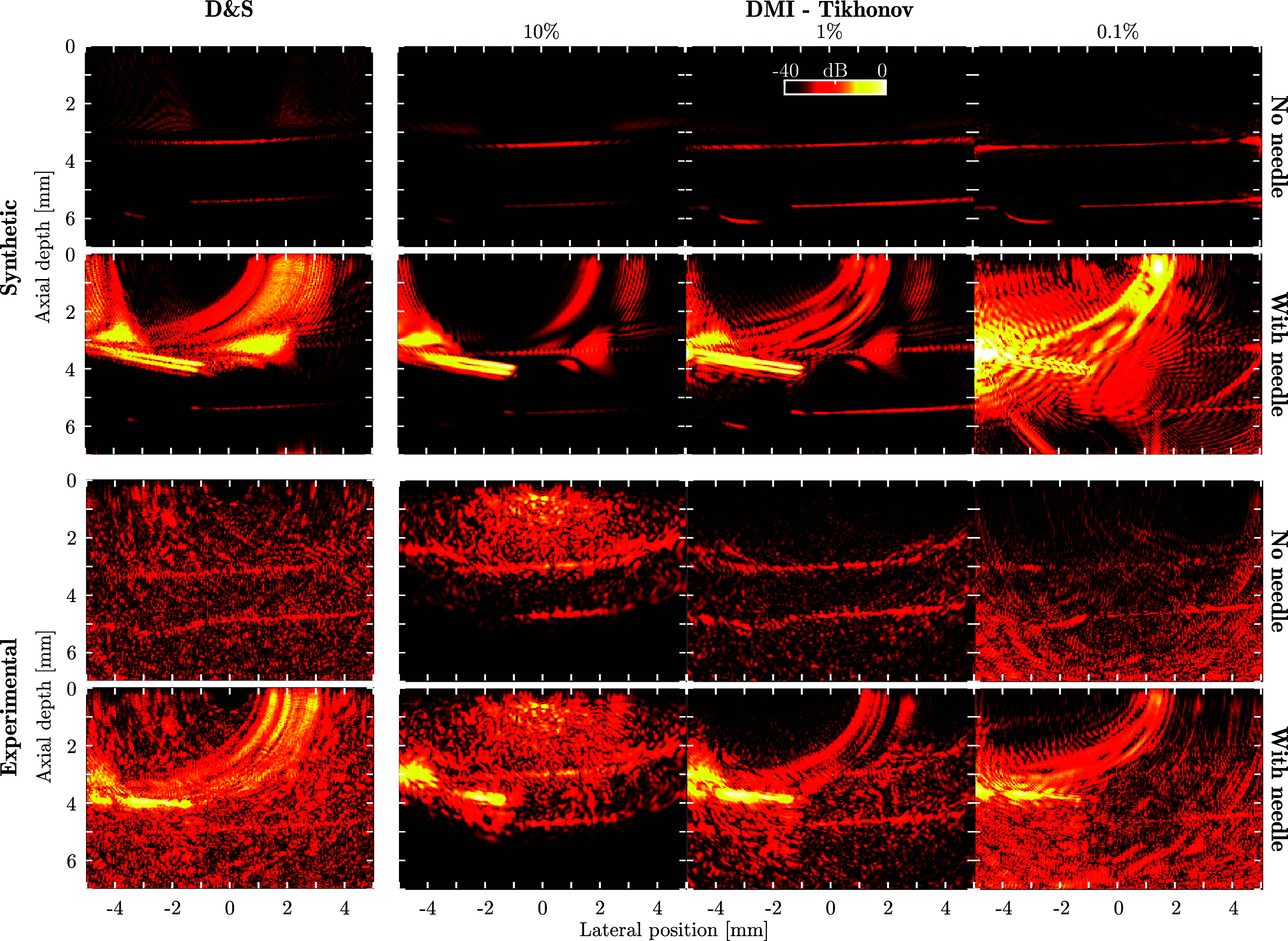
(Color online) Performance comparison between D&S (left-most column) and DMI (three right-most columns) for synthetic (top two rows) and experimental (bottom two rows) imaging data obtained during a needle insertion into the lumen of a vessel phantom. DMI reconstructions were performed under Tikhonov regularisation using α=10%, 1%, and 0.1%. All panels use the same 40 dB dynamic range.

As a final demonstration, the D&S and DMI algorithms were applied to two time frames of the needle insertion phantom, for both synthetic and experimental data (Fig. 5). Despite material inhomogeneities and (in the experimental case) a non-linear detector response, DMI again achieved substantial improvements in image quality over D&S, improving the clarity and visibility of the vessel boundary and suppressing the artefacts generated by the needle that dominated the D&S images. The value of the regularisation parameter α determines the trade-off between inversion accuracy (lower values improve the “sharpness” of the vessel boundary) and stability (inversion results at lower values suffer from increased noise). An empirically determined value of α=1% consistently resulted in the best trade-off.

The GPU-accelerated D&S images presented here required approximately 4 ms to compute, whereas the DMI images required–once the two involved matrices were computed and transferred to GPU memory–just 0.4 ms. This DMI computation time includes data transfer to and from the GPU memory. The computational performance of D&S and DMI is summarised in Table I. Note that the D&S algorithm could in principle also be converted into a matrix-vector multiplication, which might be implemented similarly efficiently via a low-rank SVD approximation.

**TABLE I.** Performance comparison between D&S and DMI. The synthetic resolutions and signal-to-clutter ratio were computed using the data in Fig. 2.

	D&S	DMI
Computation time (ms)	4.0	0.4
Corresponding frame rate (Hz)	250	2500
Memory required (GB)	0.005	5.8
Lateral resolution (*μ*m)	200	200
Axial resolution (*μ*m)	150	150
Signal-to-clutter ratio (dB)	22.1	29.4

## IV. DISCUSSION AND CONCLUSION

Based on the results presented here, DMI is an attractive alternative to the conventional D&S ultrasound image reconstruction in systems featuring low channel count. Here, applied to a freehand OpUS imaging system, DMI achieved substantial reductions in image artefacts and noise, and was able to more accurately reproduce the image amplitude, whilst producing images an order of magnitude faster than with D&S when implemented on modest hardware.

To limit computational complexity, the forward model used in this work operated under stringent assumptions. The DMI algorithm presented here was able to suppress image artefacts in the presence of material inhomogeneities–although similar spatial distortions to those observed with D&S resulted. However, the other assumptions will be harder to relax. For instance, non-linear propagation of high-intensity ultrasound cannot be incorporated in the forward linear model, and properly accounting for multiple scattering off strong contrasts or material inhomogeneity would either require accurate prior knowledge of the imaging scenario, or an iterative scheme that would hinder real-time reconstructions.

Here, only Tikhonov and truncated SVD regularisation are considered as these schemes are readily applied and result in a closed-form expression as required for rapid computations [cf. Eqs. (3) and (7)]. Furthermore, with limited additional computational cost, generalised Tikhonov regularisation could be used to, for instance, prioritise spatially smoothly varying contrast and still allow for image formation in a single iteration. However, given the sub-millisecond matrix multiplication time achieved through GPU acceleration, rapidly converging few-iteration inversion schemes could be applied instead of DMI that still achieve real-time results, but allow for a broader range of regularisation techniques, such as total variation. In addition, such few-iteration schemes could be applied to include, e.g., multiple scattering.

In this work, the forward system matrix P was generated using an impulse velocity source signature, and all sources were assumed to have identical responses. However, this resulted in the actual source signature not being inverted for, resulting in a limit of the axial resolution. In principle, the source signature of all 64 sources can be measured and incorporated, which does indeed result in improved axial resolution (compare Fig. 5 in this work with Fig. 2 of Alles *et al.*^33^) However, as the measurement of these source signatures depends on a stabilisation parameter, this signature estimation was not incorporated in this work to avoid ambiguity in the performance assessments of the DMI method.

The DMI method presented here offers substantial improvements in image quality, as well as reduced computation time, compared to D&S image reconstruction, and achieved this using modest computational hardware. In addition, the GPU-accelerated implementation allows for on-the-fly adjustment of the regularisation parameter α to tailor the DMI performance to the imaging scenario. Whilst in this work DMI was applied to an OpUS imaging setup, similar benefits are expected in other low channel-count scenarios, such as systems comprising sparse 2D or 3D imaging arrays, photoacoustic imaging, or plane wave imaging. DMI could hence simultaneously improve the image quality and reduce the experimental complexity of future ultrasound imaging systems in a wide range of settings.

## Data Availability

The data that support the findings of this study are available from the corresponding author upon reasonable request.
